# Hormonal changes of intimate partner violence perpetrators in response to brief social contact with women

**DOI:** 10.1002/ab.21995

**Published:** 2021-10-04

**Authors:** Leander van der Meij, Matias M. Pulopulos, Vanesa Hidalgo, Mercedes Almela, Marisol Lila, James R. Roney, Alicia Salvador

**Affiliations:** ^1^ Department of Industrial Engineering Eindhoven University of Technology Eindhoven The Netherlands; ^2^ Department of Experimental Clinical and Health Psychology Ghent University Ghent Belgium; ^3^ IIS Aragón, Department of Psychology and Sociology, Area of Psychobiology University of Zaragoza Teruel Spain; ^4^ Health Department Valencian International University (VIU) Valencia Spain; ^5^ Department of Cognitive Neuropsychology Tilburg University Tilburg The Netherlands; ^6^ Department of Social Psychology University of Valencia Valencia Spain; ^7^ Department of Psychological and Brain Sciences University of California Santa Barbara California USA; ^8^ Laboratory of Social Cognitive Neuroscience, IDOCAL University of Valencia Valencia Spain

**Keywords:** courtship, hormones, intimate partner violence, meta‐analysis, social contact

## Abstract

This study investigated whether men with a history of real‐life aggressive, dominant behavior show increases in testosterone and cortisol levels after brief social contact with women. Furthermore, we tested the prediction that such changes in hormones would be larger than those observed previously in young male students. Sixty‐seven male participants convicted of intimate partner violence (IPV) either had brief social contact with a female confederate (experimental condition) or a male confederate (control condition). We also performed meta‐analyses to investigate whether IPV perpetrators' hormonal responses were larger than the typical responses of young male students in prior studies. All statistical analyses were preregistered. Change in testosterone did not differ across experimental conditions, and testosterone in the IPV perpetrators actually declined from baseline in the female confederate condition. Our meta‐analysis showed that this testosterone decrease was different from the testosterone increase typically observed in young male students. The cortisol levels of IPV perpetrators did not change in response to contact with women. This result was consistent with our meta‐analysis since young male students also did not experience a cortisol change in response to interactions with women. In sum, our findings provide no evidence that male IPV perpetrators exhibit larger hormone increases to brief interactions with women, although it is possible that the men in this sample did not perceive the social contact period as a courtship opportunity. These results suggest that hormone reactivity to social encounters may differ across subject populations and depend on how subjects perceive social situations within laboratory settings.

## 1 INTRODUCTION

There is indirect evidence that testosterone and cortisol levels are relevant for mate acquisition in humans. Indeed, in young heterosexual male students, testosterone and cortisol levels increased after brief social contact with a potential mate, but not after contact with a man (testosterone: Roney et al., 2003, 2007, 2010; van der Meij et al., 2008; cortisol: Roney et al., 2007, 2010; cortisol for attractive women: van der Meij et al., 2010). Furthermore, studies have also shown that testosterone changes in response to contact with women were related to more showing‐off behaviors (i.e., courtship) during contact (Roney et al., 2007). Another study showed that increases in testosterone levels before brief social contact with women were related to more affiliative behavior (i.e., courtship) toward these women (van der Meij et al., 2012). However, currently, it is not clear if this hormonal response is universal across populations other than young male students. This study tried to address this by investigating whether the hormonal response to brief social contact with women was different in violent men compared to nonviolent men.

What could be the function of these hormonal responses to brief social contact with women? That testosterone increases after contact with women is in line with several theoretical models predicting that increased testosterone levels should facilitate the acquisition of a sexual partner. For example, the challenge hypothesis posits that testosterone increases in contexts that are relevant to human mate competition and reproduction (Archer, 2006), the Steroid/Peptide Theory of Social Bonds states that high testosterone levels relate to competition and low testosterone levels to nurturance (van Anders et al., 2011), the theoretical frameworks approach to hormones situates such tradeoffs between mate competition and other priorities as parts of broader sets of input‐mappings whereby testosterone has multiple coordinated effects on the brain and the rest of the body (Roney, 2016), and finally, testosterone has also been proposed to function as a coping mechanism in competitive situations (Salvador, 2005). Like testosterone, according to the stress literature, the hormone cortisol could also facilitate mate acquisition. Cortisol levels play a central role in the stress response (Sapolsky et al., 2000), and cortisol levels especially increase during uncontrollable socioevaluative stress (Dickerson & Kemeny, 2004). Contact with a potential romantic partner can be interpreted as such an uncontrollable socioevaluative stressor: a potential partner may reject any advances, and energy has to be mustered for impression management (van der Meij et al., 2010). Indeed, studies investigating hormonal responses to socioevaluative stress in the laboratory (Kirschbaum et al., 2008) typically show increases in cortisol (for meta‐analyses see: Goodman et al., 2017; Liu et al., 2017) and testosterone (Lennartsson et al., 2012; Phan et al., 2017; cf. Schoofs & Wolf, 2011). Alternatively, cortisol responses may be triggered even when social interactions with potential mates are not perceived as stressful since cortisol effects on energy mobilization and memory consolidation could still promote courtship efforts in such cases (see Roney et al., 2010).

Importantly, some research suggests that there may also be individual differences in the magnitude of these hormonal responses to women. One study showed that the testosterone response to brief social contact with women was larger in young male students who self‐reported to have a more aggressive dominant personality (van der Meij et al., 2008). Male students with lower numbers of CAG repeats in the androgen receptor gene—which predicts both more active androgen receptors and phenotypes associated with greater intrasexual competitiveness (Chamberlain et al., 1994; Rajender et al., 2008; Simmons & Roney, 2011)—also exhibited larger reactive testosterone increases after interactions with women (Roney et al., 2010). An interesting question is whether these larger testosterone responses in more intrasexually competitive and aggressively dominant men are also found in a group of men with a history of more extreme aggressive behaviors, such as violence against their intimate partner.

The role of testosterone in human male aggression may provide clues as to the possible role of this hormone in intimate partner violence (IPV) perpetrators. The relationship between baseline testosterone and aggressive behavior in men is not especially strong, with meta‐analyses showing significant relationships but small overall effect sizes (Archer et al., 2005). Other research, however, has found that short‐term, reactive testosterone increases may be more reliable predictors of aggressive behaviors that occur in response to social provocations (reviewed in Carré & Olmstead, 2015). Short‐term testosterone administration also triggered more aggressive behavior in men relative to placebo administration, but interestingly, only in men with high trait dominance or low self‐control (Carré et al., 2017). Testosterone has been found to reduce coupling of the orbitofrontal cortex to the amygdala (van Wingen et al., 2010), which may provide a mechanism whereby testosterone may promote aggression via the loss of frontal cortex inhibitory control of aggressive impulses that originate in subcortical structures (Mehta & Beer, 2010). Thus, current evidence suggests that reactive testosterone elevations may reduce inhibitory control of aggressive behaviors, especially among those men who already have poor impulse control and baseline tendencies toward higher dominance.

IPV offenders, almost by definition, exhibit high levels of aggressive dominance, and they have also been found to be more impulsive relative to a control sample (Romero‐Martínez et al., 2019). As such, these men may be especially susceptible to disinhibitory effects of testosterone increases on the expression of aggressive behaviors. Furthermore, such men may have qualities that predict larger testosterone elevations after interactions with women. One study showed that the testosterone response to brief social contact with women was larger in young male students who self‐reported a more aggressive dominant personality (van der Meij et al., 2008), raising the possibility that such responses would also be larger in IPV offenders. Likewise, IPV perpetrators had testosterone levels that were higher during socioevaluative stress than did men in a control sample (Romero‐Martínez, Lila, Sariñana‐González, et al., 2013) and had higher nonspecific skin conductance reactivity to social evaluative stress during the recovery period than a control sample (Romero‐Martínez, Lila, Williams, et al., 2013).

In summary, IPV offenders may be more hormonally reactive to interactions with women, and they may also respond to testosterone elevations with greater increases in aggressive behaviors in cases in which disagreements or conflicts of interest arise during the course of the interactions. The current research specifically tests the former possibility by assessing whether IPV perpetrators exhibit larger hormonal increases than nonoffenders after brief social encounters with women. Confirmation of greater hormonal reactivity could identify a physiological marker of a propensity toward IPV, which would in turn, recommend further research to test for changes in hormone responses after completion of treatment programs. To test this, we performed a study in which 34 men had brief social contact with a woman (experimental condition), and 33 men had contact with another man (control condition). Testosterone and cortisol levels were measured before and after the contact period. Furthermore, we used meta‐analytical techniques to compare effect sizes between our study and studies with young male students. All analyses were preregistered here https://osf.io/2ntcu. We tested the following hypotheses:

1. In men convicted of IPV, testosterone and cortisol levels will increase in response to brief social contact with women and remain unchanged in response to contact with men.
2. The testosterone and cortisol change in response to brief social contact with women will be larger in men convicted of IPV than in young male students.

## 2 METHODS

### 2.1 Participants

Sixty‐seven men convicted of domestic violence participated in this study (age years: *M* = 41, SD = 12, range = 21–76). They were recruited from a psychoeducational and community‐based treatment program (*Contexto programme*) of the University of Valencia (Lila et al., 2013, 2018) before starting their treatment. Participants in this program had been sentenced to less than 2 years in prison for IPV. They could be classified as Type II batterers (Gottman et al., 1995) because the violence against their partner was impulsive and not premeditated. The program mainly consisted of several group sessions with the aim to reduce risk factors and increase protective factors for violent behavior against women in intimate relationships (for details, see Lila et al., 2013). Criteria for exclusion were: (i) a previous criminal record, (ii) a serious mental disorder, (iii) a serious addiction to alcohol or other substances. Attendance at this program led to the suspension of their sentence.

The participants were randomly assigned to one of the two conditions (contact with a woman: *n* = 34, contact with a man: *n* = 33). There were no significant differences between the two groups in age, educational level, alcohol consumption, and number of cigarettes smoked per day (see Table 1). However, the participants in the condition that had contact with a woman reported lower subjective socioeconomic status (Adler et al., 2000), had slightly lower body mass index, and the number of participants regularly taking drugs was slightly higher than in the group that had contact with a man, see Table 1.

**Table 1:** Characteristics of the participants who had contact with a woman (*n* = 34) or man (*n* = 33)

	Contact with women		Contact with men				
	*M*	SD	*M*	SD	*t/χ* ^2^	*df*	*p*
Age (years)	40.62	12.54	41.00	10.80	−0.13	65	.894
SES	3.53	1.71	4.55	1.42	−2.65	63.45	.010
BMI	24.96	3.17	27.97	9.48	−1.73	38.86	.091
Education					2.78	4	.596
None	1		2				
Primary	22		15				
Secondary	8		12				
Vocational	1		2				
University	2		2				
Daily alcohol units	4.06	5.30	5.24	8.51	−0.69	65	.495
Daily cigarettes	8.91	9.41	7.37	7.95	0.72	64	.475

	Contact with women		Contact with men				
	Yes	No	Yes	No	*t/χ* ^2^	*df*	*p*
Regular recreational drugs	10	24	4	29	3.03	1	.082
Antidepressants and benzodiazepines	6	28	3	30	1.05	1	.305
In a relationship	13	21	16	17	0.72	1	.397

*Note*: Regular recreational drugs: daily use of marihuana, or weekly use of 3.5 g of marihuana, or once a month or more: ecstasy, amphetamines, cocaine, and hallucinogens.

Abbreviations: BMI, body mass index; SES, subjective socioeconomic status.

All the participants reported being heterosexual (open question: what is your sexual orientation?). None of the participants met any of three of the four criteria for exclusion as specified in the preregistration of our analyses, see here https://osf.io/2ntcu. These three criteria were: (i) medication that directly affects the secretagogues of the hypothalamic–pituitary–adrenal axis or hypothalamic–pituitary–gonadal axis, (ii) recovery from severe medical treatment (e.g., chemotherapy, heart surgery), (iii) under the influence of drugs or alcohol during the study. However, we found that nine participants met a fourth exclusion criteria, as they probably took daily medication (exact frequency unspecified) that could affect cognitive, behavioral, or emotional responses (either antidepressants or benzodiazepines). However, excluding such a significant percentage of participants from our analyses would substantially reduce the power to detect an effect. Therefore, we chose to report our results with and without these participants and include this medication use as a covariate, see Table S1 in the Supporting Information.

### 2.2 Procedure

The experiment was carried out at the Faculty of Psychology (University of Valencia). To recruit participants, during the first session of the psychoeducational and community‐based treatment program (*Contexto*), they were informed that we were looking for volunteers for a study investigating the physiological changes during the performance of some tasks. To strengthen our cover story during the interaction with the stimulus person, we told them that we were looking for volunteers among all the people who come to the faculty. If they agreed to participate, the men would participate in our study before starting their psychoeducational treatment program.

Before starting the study, participants were instructed to refrain from alcohol consumption and any heavy physical activity on the day of the study and the day before it. Furthermore, during the 2 h immediately before the session, participants were asked to drink only water and avoid any stimulants, such as coffee, cola, caffeine, tea, or chocolate. Upon arrival at the laboratory, the participants were briefed on the general procedure of the study. All participants received verbal information about the study and signed an informed consent form about the general procedure and the measurements taken. Participants were not informed that they would have to wait for 10 min in the same room with another individual as part of this study.

The session started with 20 min of habituation in a quiet room (room A). During this phase, their height and weight were measured, and the participants were asked to complete a questionnaire. At the end of the habituation, the participants provided the first saliva sample (Sample 1) to measure their cortisol and testosterone levels. Participants were then brought into a different room (room B) where there was a confederate present who appeared to be another participant of the study. As part of the cover story, the experimenter informed the participant and the stimulus person that he had to make some extra copies of the questionnaires, and the participant and stimulus person were then asked to wait. The participant and the stimulus person were left alone to wait together for 10 min. After 10 min, the experimenter returned to room B and asked the stimulus person to leave the room. Then, the participant completed two questionnaires for 10 min. Finally, the participant was brought into room A, where he was asked to provide another salivary sample (Sample 2) and complete some questionnaires. The second sample was 20 min after the start of the conversation, which was the same as the other studies in the meta‐analyses (Roney et al., 2007; van der Meij et al., 2010), except for the Roney et al. (2010) study, which was 40 min after the conversation. Finally, participants were debriefed about the true nature of the experiment and received €10. To control for the circadian rhythm of cortisol, all the sessions started between 4:00 and 5:00 p.m., and there were no differences between groups in starting time (*p* = .816).

The study was approved by the ethical committee of the Faculty of Psychology (University of Valencia) and was conducted in accordance with the Declaration of Helsinki.

### 2.3 Stimulus persons

Four men (age: 27, 29, 29, and 30 years) and six women (age: 22, 23, 27, 28, 31, and 33 years) played the role of the stimulus person. The confederates were researchers from our laboratory and did not have contact with the participant before the experiment. Following van der Meij et al. (2012) and Roney et al. (2003), the confederates were instructed to engage in a friendly conversation in a natural manner and were asked to act as if they were participants in the same study and to allow long pauses if the participants elected not to talk. On average participants rated the female confederates as just above average on attractiveness (*M* = 5.06, SD = 1.65) and male confederates were rated as below average on attractiveness (*M* = 3.39, SD = 3.39) on a scale from 1 (not very attractive) to 7 (very attractive). The research assistant carrying out the study was male.

### 2.4 Hormonal assays

Salivary samples to assess cortisol and testosterone levels were collected by passive drool. Participants were asked to deposit 5 ml of saliva in plastic vials, and samples were frozen at −80°C. Biochemical analyses were done by the Laboratory of Social Cognitive Neuroscience (Faculty of Psychology at the University of Valencia). For cortisol levels, the samples were analyzed in duplicate with the salivary cortisol enzyme‐immunoassay kit from Salimetrics. Assay sensitivity was 0.007 μg/dl. For each subject, all the samples were analyzed in the same trial. The mean inter‐ and intra‐assay coefficients of variations were all below 10%. For testosterone levels, the samples were analyzed in duplicate using enzyme‐immunoassays with the expanded range salivary testosterone enzyme‐immunoassay kit from Salimetrics. Assay sensitivity was 1.0 pg/ml, and the mean inter‐ and intra‐assay coefficients of variation were all below 10%.

### 2.5 Statistical analyses Hypothesis 1

The statistical analyses to test Hypothesis 1 were preregistered here https://osf.io/2ntcu. To investigate Hypothesis 1, we used linear mixed models for repeated measures in SPSS (one analysis for each hormone). As the dependent variable, we included either the testosterone or cortisol levels. As predictors, we included Moment (pre or post) and Condition (contact with men or women). We selected an unstructured correlation metric as the covariance structure.

*Robustness statistical analyses Hypothesis 1*: We also tested how robust statistical conclusions were when analyzing Hypothesis 1, see Table 1. To this end, we investigated whether the statistical conclusions differed when including several control variables, see Table 1 (covariates were added one at a time). Additionally, we investigated whether the statistical conclusions were different when excluding outliers. We followed the guidelines by Pollet and van der Meij (2017) for outlier detection and detected two testosterone outliers (same participant) and one cortisol outlier (all ≥3 SD from the mean). There were no outliers detected using the three or more interquartile ranges above the third quartile or below the first quartile. One measurement error (value was 0) was detected in the hormone cortisol, and this value was removed. Testosterone and cortisol levels were log‐transformed for all analyses (this is a common practice in hormonal research) as their skewness was larger than 1. In the Supporting Information, we also tested our results' robustness by performing the analyses with the raw scores, and we report there the absolute changes in hormonal levels.

Furthermore, we tested how much confidence should be placed in our result using Bayesian analyses for paired samples. These analyses were performed using JASP version 0.14.1 with default prior scales, following van Doorn et al. (2020).

### 2.6 Statistical analyses Hypothesis 2

The statistical analyses to test Hypothesis 2 were preregistered here https://osf.io/2ntcu. To test Hypothesis 2, we used meta‐analyses with the package Metafor (Viechtbauer, 2010) in R. We used a restricted maximum likelihood estimator for heterogeneity, as this estimator shows a good balance between unbiasedness and efficiency (Viechtbauer, 2005). In the meta‐analysis, we included for each study the pre and post means (log‐transformed) with their corresponding standard deviation (log‐transformed), sample size, and the correlation coefficient between measures. As effect size, we used the standardized mean change using raw score standardization. To investigate if the change between Time 2 and Time 1 in this study was larger than the testosterone change of other studies, we included the moderator “Population” (1 = IPV perpetrators, 0 = young male students). We compared the current population of IPV perpetrators with other studies that tested hormonal changes in response to brief social contact with women. We identified the following studies: (i) Roney et al. (2003, *n* = 19), (ii) Roney et al. Study 1 (2007, *n* = 77), (iii) Roney et al. Study 2 (2007, *n* = 46), (iv) van der Meij et al. (2008, *n* = 30), (v) Roney et al. (2010, *n* = 90), see also the preregistration. We also added an unpublished study from the first author with the same methodology (not included in original preregistration, *n* = 21). The total sample size of the testosterone meta‐analysis was 315.

We also used the same meta‐analytic approach to compare if the cortisol change in the sample of IPV perpetrators was larger than the cortisol change in young male students in the following studies: (i) Roney et al. Study 1 (2007, *n* = 77), (ii) Roney et al. Study 2 (2007, *n* = 42), (iii) Roney et al. (2010, *n* = 82). We also added an unpublished study from the first author (*n* = 21) and the study from van der Meij et al. (2010, *n* = 40) with the same methodology (both were omitted from the original preregistration). The total sample size of the cortisol meta‐analysis was 293.

All tests were two‐tailed, and we considered *p*‐values smaller than an *α*‐level of 0.050 as statistically significant. We checked for publication bias with Duval and Tweedie's trim and fill (Duval & Tweedie, 2000) and used a regression test to investigate asymmetry in the funnel plot. We also tested if using the raw hormonal values instead of the log‐transformed values changed the meta‐analyses' statistical conclusions (see also the Supporting Information).

## 3 RESULTS

### 3.1 Hypothesis 1: Testosterone

**Figure 1:**
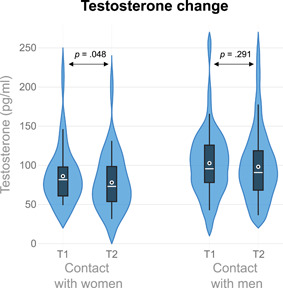
Violin plot of the absolute testosterone levels per Moment and Condition. *p* Values refer to the comparisons made within the mixed model using the log‐transformed values. The plot used an Epanechnikov kernel. White circle = mean; white line = median [Color figure can be viewed at wileyonlinelibrary.com]

The mixed model with testosterone as the dependent variable showed that there was no interaction between Moment and Condition (*F*_1, 62.20_ = 0.43, *p* = .515, *η*_p_^2^ = 0.01). However, there was a main effect of Moment (*F*_1, 62.20_ = 4.72, *p* = .034, *η*_p_^2^ = 0.07), which showed that testosterone levels decreased after social contact irrespective of the sex of the stimulus person (*d*_rm_ = 0.21). Although there was no significant interaction between Moment and Condition we did analyze the testosterone response separately by condition to explore potential trends in line with our hypothesis. These results showed that testosterone levels decreased after contact with women (*t*_62.18_ = 2.04, *p* = .048, *d*_rm_ = 0.20) and did not change after contact with men (*t*_62.22_ = 1.07, *p* = .291, *d*_rm_ = 0.10). Furthermore, there was a marginally significant effect of Condition (*F*_1, 64.60_ = 3.73, *p* = .058, *η*_p_^2^ = 0.05), showing that the participants who had contact with women had overall lower testosterone levels than participants who had contact with men (*d* = 0.34). Adding covariates and removing outliers in the mixed model did not substantially change the statistical conclusions of the testosterone decrease in response to women, but when only using raw testosterone values, the change in testosterone was nonsignificant after contact with women (*t*_61.13_ = 1.61, *p* = .112, *d*_rm_ = 0.13); see also the Supporting Information. Furthermore, Bayesian analyses showed strong evidence for the absence of an increase in testosterone levels (H0) in response to women (BF_10_ = 0.06) compared to an increase in testosterone levels (H1). Furthermore, there was moderate evidence (4.93 times more likely) for testosterone levels to decline after contact with women (H1) compared to testosterone levels not declining (H0). See Figure 1 for the raw testosterone means per Condition and Moment.

### 3.2 Hypothesis 1: Cortisol

**Figure 2:**
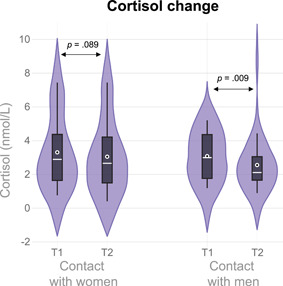
Violin plot of the absolute cortisol levels per Moment and Condition. *p* Values refer to the comparisons made within the mixed model using the log‐transformed values. The plot used an Epanechnikov kernel, White circle = mean; white line = median [Color figure can be viewed at wileyonlinelibrary.com]

The mixed model with cortisol as dependent variable showed that there was no interaction between Moment and Condition (*F*_1, 60.26_ = 0.498, *p* = .483, *η*_p_^2^ = 0.01) and no main effect of Condition (*F*_1, 64.31_ = 0.10, *p* = .749, *η*_p_^2^ < 0.01). However, there was a main effect of Moment (*F*_1, 60.26_ = 9.81, *p* = .003, *η*_p_^2^ = 0.14), showing that cortisol levels decreased after social contact irrespective of the sex of the stimulus person (*d*_rm_ = 0.28). Although there was no significant interaction between Moment and Condition we did analyze the cortisol response separately by condition to explore potential trends in line with our hypothesis. These results showed that cortisol levels decreased after contact with men (*t*_60.28_ = 2.72, *p* = .009, *d*_rm_ = 0.24) and did not change after contact with women (*t*_60.23_ = 1.72, *p* = .089, *d*_rm_ = 0.15). Adding covariates, removing outliers, and only using raw cortisol values did not change the statistical conclusions of the cortisol decrease in response to women (see the Supporting Information). Furthermore, Bayesian analyses showed strong evidence for the absence of an increase in cortisol levels (H0) in response to women (BF_10_ = 0.09) compared to an increase in cortisol levels (H1). See Figure 2 for the raw cortisol means per Condition and Moment.

### 3.3 Hypothesis 2: Meta‐analyses testosterone

**Figure 3:**
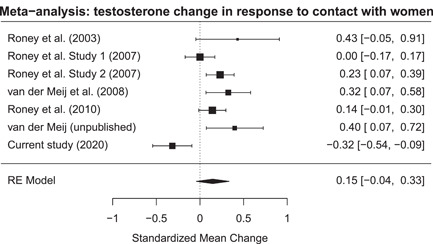
Forest plot of all the studies measuring a testosterone change in response to contact with women

*All studies*. The meta‐analysis showed that when including all studies there was no change in testosterone levels in response to contact with women (*k* = 7, estimate = 0.15, SE = 0.10, *z* = 1.53, *p* = .127, 95% CI [−0.04 to 0.33]). However, effect size variance could be explained by between study differences (*τ* = 0.22, *τ* ^2^ = 0.05, *I* ^2^ = 80.15%, *H* ^2^ = 5.04, *Q*_6_ = 25.25, *p* < .001). Including the moderator showed that the testosterone change was smaller in IPV perpetrators than in young male students (*Q*_1_ = 9.86, *p* = .002; estimate = 0.52, SE = 0.16, *z* = 3.14, 95% CI [0.19–0.84]), see Figure 3. The statistical conclusions of this meta‐analysis remained the same when using the raw testosterone values (see the Supporting Information).

*Excluding data from the current study*. Not including the data on the IPV perpetrators showed that testosterone levels increased in response to contact with women (*k* = 6, estimate = 0.20, SE = 0.06, *z* = 3.20, *p* = .001, 95% CI [0.08–0.32]). Additionally, effect size variance could not be explained by between study differences (*τ* = 0.10, *τ* ^2^ = 0.01, *I* ^2^ = 46.18%, *H* ^2^ = 1.86, *Q*_5_ = 8.93, *p* = .112). Inspection of the forest plot did not reveal any outliers. Evidence concerning the presence of a publication bias was mixed: there was no asymmetry in the funnel plot (*t*_4_ = 1.83, *p* = .14) but Duval and Tweedie's trim and fill approach revealed that two studies could be filled below the estimated effect size. Addition of these two studies resulted in a slightly smaller overall effect size (estimate = 0.16, SE = 0.06, *z* = 2.75, *p* = .006, 95% CI [0.05–0.27]).

### 3.4 Hypothesis 2: Meta‐analyses cortisol

**Figure 4:**
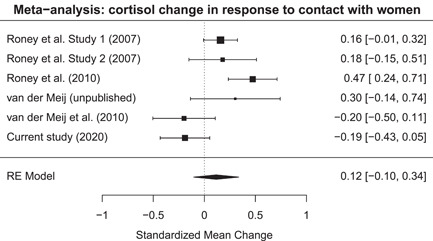
Forest plot of all the studies measuring a cortisol change in response to contact with women

*All studies*. The meta‐analysis showed that when including all studies there was no cortisol change in response to contact with a woman (*k* = 6, estimate = 0.12, SE = 0.11, *z* = 1.03, *p* = .301, 95% CI [−0.10 to 0.34]). However, effect size variance could be explained by between study differences (*τ* = 0.23, *τ* ^2^ = 0.05, *I* ^2^ = 75.65%, *H* ^2^ = 4.11, *Q*_5_ = 19.92, *p* = .001). Including the moderator showed the cortisol change in IPV perpetrators was not different from young male students (*Q*_1_ = 2.01, *p* = .156; estimate = 0.37, SE = 0.26, *z* = 1.42, 95% CI [−0.14 to 0.89]), see Figure 4. The statistical conclusions of this meta‐analysis remained the same when using the raw cortisol values (see the Supporting Information).

*Excluding data from the current study*. Not including the data on the IPV perpetrators showed that cortisol levels did not change in response to contact with women (*k* = 5, estimate = 0.18, SE = 0.11, *z* = 1.64, *p* = .102, 95% CI [−0.04 to 0.41]). However, effect size variance could be explained by between study differences (*τ* = 0.20, *τ* ^2^ = 0.04, *I* ^2^ = 68.88%, *H* ^2^ = 3.21, *Q*_4_ = 12.07, *p* = .017). Inspection of the forest plot did not reveal any outliers. There was also no evidence for publication bias as there was no asymmetry in the funnel plot (*t*_3_ = −0.12, *p* = .913) and Duval and Tweedie's trim and fill approach revealed that no studies could be filled below or above the estimated effect size.

## 4 DISCUSSION

Our results concerning testosterone did not confirm our predictions: testosterone responses did not differ across the female and male confederate conditions, and in the female condition specifically, the testosterone levels of IPV perpetrators declined from baseline. Our meta‐analysis also showed that this decrease was different from young male students as the latter typically showed an increase in testosterone levels in response to social contact with women. These results were surprising as we had hypothesized that perpetrators would respond with an even higher testosterone response than young male students. We based our prediction on previous findings showing that a more self‐reported aggressive dominant personality in young male students was related to a larger testosterone increase in response to brief social contact with women (van der Meij et al., 2008). Additionally, compared to a control group, IPV perpetrators experienced higher testosterone levels during socioevaluative stress (Romero‐Martínez, Lila, Sariñana‐González, et al., 2013), had longer psychophysiological activation after stress (Romero‐Martínez, Lila, Williams, et al., 2013), and were more impulsive than healthy controls (Romero‐Martínez et al., 2019). The current results do not support the use of reactive testosterone response as a physiological marker of a propensity toward IPV.

Why was the testosterone response so different between populations? We think the most likely explanation may be that, even though the IPV perpetrators rated the women with whom they had contact as just above average on attractiveness, they may not have seen these women as a potential sexual partner. First, the participants were considerably older than the women with whom they had contact. Second, their socioeconomic background was lower. On average, the IPV perpetrators self‐reported a 3.5 on the socioeconomic status ladder (Adler et al., 2000), which can be considered low (1 = lowest, 10 = highest). In addition, only 2 of the 34 men who had contact with a woman had a university degree, whereas all the stimulus persons were university students. In conclusion, the women with whom they had contact were perhaps dissimilar from women they would typically date, and thus they did not put effort into impression management. Additionally, participating in this study may have been too novel and unfamiliar for participants even to consider an opportunity for courtship. Unlike student populations, most of the men convicted of IPV were unfamiliar with a university setting and participating in an experiment. In sum, how participants perceived the contact period might have been more important than our theoretical rationale in the introduction and the indirect evidence from previous studies (Romero‐Martínez et al., 2019; Romero‐Martínez, Lila, Sariñana‐González, et al., 2013; Romero‐Martínez, Lila, Williams, et al., 2013; van der Meij et al., 2008). Nonetheless, a limitation of the meta‐analysis is that the true effect size for testosterone responses in male students may turn out to be smaller with the addition of more studies. If such studies would be reported, then it may be that the perpetrators do not differ from students.

Why did testosterone levels decrease throughout the study and not remain stable? We think the most likely explanation for this finding is that testosterone levels were elevated in anticipation of the study. Participants knew that by participating, they were going to be evaluated and observed. Theoretical models such as the challenge hypothesis (Archer, 2006) predict that, in such situations where social status is at stake, testosterone levels increase. Indeed, testosterone levels can increase in anticipation of an event (Salvador et al., 2003), dyadic intrasexual competition increases testosterone levels (Kordsmeyer & Penke, 2019), and socioevaluative stress tasks in the laboratory typically produce an increase in testosterone levels (Lennartsson et al., 2012; Phan et al., 2017; cf. Schoofs & Wolf, 2011). During participation in the study, participants may have realized that this evaluative component was not that important, leading to a decrease in arousal and testosterone levels.

The results concerning cortisol also show an overall decrease throughout the study, and this thus also shows that cortisol levels may have been elevated in anticipation of the study. However, although there was no statistically significant difference between conditions, it is interesting that we found no change in cortisol levels in response to contact with women. This last result was more in line with the literature since our meta‐analysis showed that, also for young male students, there was no statistically significant change in cortisol levels in response to contact with women. However, it should be noted that the meta‐analysis was based on a small number of studies and found a small effect size in the predicted direction. Furthermore, when excluding the studies not included in the original preregistration, there was actually a statistically significant change in cortisol levels (see the Supporting Information). Interestingly, some preliminary evidence shows that the cortisol response toward women is moderated by other factors, which could reduce the power to detect an effect. For example, men with more sexual experience showed higher testosterone responses in one study (Roney et al., 2003), although follow‐up studies failed to replicate this result (Roney et al., 2007; van der Meij et al., 2008). Also, men responded with a bigger increase in cortisol levels when they perceived the woman with whom they had contact as attractive (van der Meij et al., 2010). Thus, more data from future studies may be needed to clarify if there is a cortisol response toward potential mates in men.

This study also had several limitations that are mainly related to the unique sample. A first limitation is the modest sample size. Studies with a bigger sample size may show that the hormonal response of IPV perpetrators to women or men may differ. A second limitation is that other conditions could have been included that would have strengthened the experimental evidence. Possibilities could be a condition in which participants: (i) had contact with a stimulus person who was matched on age and socioeconomic status, (ii) only waited and had no social contact, (iii) were not convicted of domestic violence. A final limitation is that relatively many of our participants used regular recreational drugs (slightly more participants who had contact with women) and antidepressants & benzodiazepines. This may cause hyperprolactinemia and indirectly affect the luteinizing hormone releasing (LNRH) axis, which could have influenced the participants' hormonal response. Although it may be difficult, including more intimate violence perpetrators without this drug use would improve result robustness. It would be a real challenge for future research to address all previously mentioned limitations as it would mean increasing the sample size substantially. In the current study, it was already complicated to get the current number of participants who had a history of domestic violence.

In conclusion, this study did not find any evidence that male IPV perpetrators exhibit larger hormone responses to social interactions with women than other men, despite sound theoretical reasons to predict this possibility. Although it is possible that perpetrators might exhibit larger hormone responses under circumstances in which women interaction partners are more similar to them in age and socioeconomic status, our findings nonetheless argue against robust hormone reactions to interactions with women in general, even though the perpetrators rated the women interactants as above average in attractiveness. Concerning the hormone responses to social interactions among all men, our findings provide evidence that findings may importantly differ across different subject populations. Our meta‐analysis indicated that young male students exhibited larger testosterone responses to women than did this sample's IPV perpetrators, suggesting that the hormone responses may be moderated by variables such as viewing the interaction as a courtship opportunity or perhaps even by age itself. Such moderators could be more rigorously tested in future research on hormonal responses to interactions with potential mates.

## Supporting information

Supporting information.

## Data Availability

The data that support the findings of this study are available from the corresponding author upon reasonable request.
