# Supplementary material for: Hormonal changes of intimate partner violence perpetrators in response to brief social contact with women
Source: Aggress Behav. 2021 Oct 4;48(1):30–9. doi: 10.1002/ab.21995 (PMC9293448; doi:10.1002/ab.21995)
Supplement: Supplementary file 1 — Supporting information. [file AB-48-30-s001.docx]

**Supplementary analyses: intimate partner violence perpetrators**

**Absolute values of hormonal levels**

*Table S1:* Mean and SD of testosterone (pg/ml) and cortisol (nmol/L) levels across conditions

|  | Contact with women | | Contact with men | |
| --- | --- | --- | --- | --- |
|  | *Mean* | *SD* | *Mean* | *SD* |
|  |  |  |  |  |
| Testosterone T1 | 86.21 | 35.76 | 102.86 | 45.63 |
| Testosterone T2 | 78.13 | 36.64 | 98.23 | 45.87 |
| Cortisol T1 | 3.31 | 2.18 | 3.08 | 1.44 |
| Cortisol T2 | 3.05 | 2.19 | 2.55 | 1.54 |

*Table S2:* Mean and SD of Δ the change in testosterone (pg/ml) and cortisol (nmol/L) levels

|  | *Mean* | *SD* |
| --- | --- | --- |
|  |  |  |
| Δ Change Testosterone | -7.38 | 25.80 |
| Δ Change Cortisol | -.48 | 1.31 |

*Table S3:* The skewness statistic of before and after log transforming the hormonal values

|  | Before log  transforming | | After log  transforming | | |
| --- | --- | --- | --- | --- | --- |
|  | *Statistic* | *s.e.* | *Statistic* | *s.e.* |  |
|  |  |  |  |  |  |
| Testosterone T1 | 1.41 | .30 | -.36 | .30 |  |
| Testosterone T2 | 1.09 | .29 | -.36 | .29 |  |
| Cortisol T1 | .71 | .31 | -.67 | .31 |  |
| Cortisol T2 | 1.29 | .29 | -.60 | .29 |  |

**Adding covariates and exclusion criteria**

*Table S4:* How *p* values and effect sizes change for testosterone and cortisol responses after contact with women when controlling for covariates and excluding participants.

|  | **Testosterone change**  **contact women** | | **Cortisol change**  **contact women** | |
| --- | --- | --- | --- | --- |
|  | *d_rm_* | *p* | *d_rm_* | *p* |
|  |  |  |  |  |
| Original result | .20 | .048 | .15 | .089 |
|  |  |  |  |  |
| **Excluding** |  |  |  |  |
| Anti-depressants & benzodiazepines | .17 | .098 | .15 | .090 |
| Outliers | .21 | .050 | .16 | .087 |
|  |  |  |  |  |
| **Covariates** |  |  |  |  |
| Anti-depressants & benzodiazepines | .20 | .049 | .15 | .088 |
| Daily alcohol intake | .20 | .049 | .16 | .086 |
| Daily smoked cigarettes | .20 | .049 | .15 | .099 |
| Regular recreational drugs | .20 | .047 | .15 | .100 |
| Medical condition affecting behavior | .19 | .049 | .15 | .087 |
| Medication indirectly affecting hormones | .20 | .047 | .16 | .087 |

Note: Regular recreational drugs (1 = yes, 0 = no): once a month or more: ecstasy/ amphetamines/cocaine/ hallucinogens, or daily use of marihuana, or weekly use of 3.5 grams of marihuana. Medical condition affecting behavior (1 = yes, 0 = no): medication taken on a weekly basis that can affect cognitive and emotional responses, or suffering from a mental disease that can affect cognitive, behavioral, or emotional responses. Medication indirectly influencing hormonal levels (1 = yes, 0 = no): medication use that has the potential to indirectly influences HPA and HPG axis functioning, or non-frequent (weekly or less) minor doses of oral/nasal/topical corticosteroids, or medications with the potential to influence salivary composition and availability indirectly by affecting the activity of the sympathetic or parasympathetic nervous system.

**Results hypotheses 1 without log transforming the hormonal levels**

*Testosterone raw values*

For these analyses we used the raw testosterone values and did not log transform testosterone. The mixed model with testosterone as the dependent variable showed that there was no interaction between Moment and Condition (*F*_1,61.14_ = <.01, *p* = .945, η_p_^2^ < .01). However, there was a main effect of Moment (*F*_1,61.14_ = 4.80, *p* = .032, η_p_^2^ = .07), which showed that testosterone levels decreased after social contact irrespective of the sex of the stimulus person (*d_rm_* = .17). Separating by condition showed that testosterone levels did not change after contact with women or men (respectively: *t*_61.13_ = 1.61, *p* = .112, *d_rm_* = .13; *t*_61.15_ = 1.49, *p* = .142, *d_rm_* = .12). Furthermore, overall testosterone levels were different across Condition (*F*_1,64.30_ = 4.23, *p* = .044, η_p_^2^ = .06), showing that the participants who had contact with women had overall lower testosterone levels than participants who had contact with men (*d* = .36).

*Cortisol raw values*

For these analyses we used the raw cortisol values and did not log transform cortisol. The mixed model with cortisol as dependent variable showed that there was no interaction between Moment and Condition (*F*_1,58.50_ = 2.21, *p* = .142, η_p_^2^ = .04) and no main effect of Condition (*F*_1,62.63_ = .317, *p* = .576, η_p_^2^ = .01). However, there was a main effect of Moment (*F*_1,58.50_ = 7.95, *p* = .007, η_p_^2^ = .12), showing that cortisol levels decreased after social contact irrespective of the sex of the stimulus person (*d_rm_* = .25). Separating by condition showed that, cortisol levels decreased after contact with men (*t*_58.52_ = 3.02, *p* = .004, *d_rm_* = .27) and did not change after contact with women (*t*_60.23_ = .95, *p* =.346, *d_rm_* = .08).

**Supplementary analyses: meta-analyses**

**Only including the preregistered studies**

*Meta-analyses testosterone preregistered studies only*

*All studies* The meta-analysis showed that when including all studies there was no change in testosterone levels in response to contact with women (*k*  = 6, *estimate* = .11, *se* = .10, *z* = 1.10, *p* = .273, 95% CI [-.09, .31]). However, effect size variance could be explained by between study differences (*τ* = .22, *τ^2^* = .05, *I^2^* = 81.37%, *H^2^* = 5.37, *Q*_5_ = 22.22, *p* < .001). Including the moderator showed that the testosterone change was smaller in intimate partner violence perpetrators than young male students (*Q*_1_ = 9.66, *p* = .002; *estimate* = .49, *se* = .16, *z* = 3.11, 95% CI [.18, .80]), see Figure S1.

*Excluding data current study* Not including the data on the intimate partner violence perpetrators showed that testosterone levels increased in response to contact with women (*k*  = 5, *estimate* = .18, *se* = .06, *z* = 2.80, *p* = .005, 95% CI [.05, .30]). Additionally, effect size variance could not be explained by between study differences (*τ* = .09, *τ^2^* = .01, *I^2^* = 43.42%, *H^2^* = 1.77, *Q*_4_ = 7.03, *p* = .134). Inspection of the forest plot did not reveal any outliers. Evidence concerning the presence of a publication bias was mixed: there was no asymmetry in the funnel plot (*t*_3_ = 1.22, *p* = .310) but Duval and Tweedie’s trim and fill approach revealed that two studies could be filled below the estimated effect size. Addition of these two studies resulted in a slightly smaller overall effect size (*estimate* = .13 *se* = .06, *z* = 2.09, *p* = .036, 95% CI [.01, .25]).

**Figure S1:** Forest plot of the preregistered studies measuring a testosterone change in response to contact with women.

*Meta-analyses cortisol preregistered studies only*

*All studies* The meta-analysis showed that when including all studies there was no cortisol change in response to contact with a woman (*k*  = 4, *estimate* = .16, *se* = .14, *z* = 1.13, *p* = .257, 95% CI [-.11 .42]). However, effect size variance could be explained by between study differences (*τ* = .24, *τ^2^* = .06, *I^2^* = 80.74%, *H^2^* = 5.19, *Q*_3_ = 14.67, *p* = .002). Including the moderator showed the cortisol change in intimate partner violence perpetrators was different from young male students (*Q*_1_ = 4.58, *p* = .032; *estimate* = .46, *se* = .21, *z* = 2.14, 95% CI [.04, .88]), see Figure S2.

*Excluding data current study* Not including the data on the intimate partner violence perpetrators showed that cortisol levels increased in response to contact with women (*k*  = 3, *estimate* = .27, *se* = .11, *z* = 2.52, *p* = .012, 95% CI [.06, .48]). Furthermore, effect size variance could not be explained by between study differences (*τ* = .14, *τ^2^* = .02, *I^2^* = 57.05%, *H^2^* = 2.33, *Q*_2_ = 4.73, *p* = .094). Inspection of the forest plot did not reveal any outliers. There was also no evidence for publication bias as there was no asymmetry in the funnel plot (*t*_1_ = .37, *p* = .773) and Duval and Tweedie’s trim and fill approach revealed that no studies could be filled below or above the estimated effect size.

**Figure S2:** Forest plot of the preregistered studies measuring a cortisol change in response to contact with women.

**Meta-analyses with only raw hormonal values (without log transformation)**

*Meta-analyses raw testosterone values*

*All studies raw testosterone values* The meta-analysis showed that when including all studies there was no change in testosterone levels in response to contact with women (*k*  = 7, *estimate* = .14, *se* = .09, *z* = 1.59, *p* = .112, 95% CI [-.03, .31]). However, effect size variance could be explained by between study differences (*τ* = .20, *τ^2^* = .04, *I^2^* = 79.24%, *H^2^* = 4.82, *Q*_6_ = 27.56, *p* < .001). Including the moderator showed that the testosterone change was smaller in intimate partner violence perpetrators than young male students (*Q*_1_ = 4.56, *p* = .033; *estimate* = .39, *se* = .18, *z* = 2.14, 95% CI .03, .75]), see Figure S3.

*Excluding data current study raw testosterone values* Not including the data on the intimate partner violence perpetrators showed that testosterone levels increased in response to contact with women (*k*  = 6, *estimate* = .20, *se* = .08, *z* = 2.60, *p* = .009, 95% CI [.05, .34]). Additionally, effect size variance could be explained by between study differences (*τ* = .14, *τ^2^* = .02, *I^2^* = 65%, *H^2^* = 2.86, *Q*_5_ = 13.49, *p* = .019). Inspection of the forest plot did not reveal any outliers. Evidence concerning the presence of a publication bias was mixed: there was no asymmetry in the funnel plot (*t*_4_ = .93, *p* = .407) but Duval and Tweedie’s trim and fill approach revealed that two studies could be filled below the estimated effect size. Addition of these two studies resulted in a non-significant change in testosterone after contact with women (*estimate* = .12, *se* = .07, *z* = 1.71, *p* = .088, 95% CI [-.02, 27]).

**Figure S3:** Forest plot of all the studies measuring a raw testosterone change in response to contact with women.

*Meta-analyses raw cortisol values*

*All studies* *raw cortisol values* The meta-analysis showed that when including all studies there was no cortisol change in response to contact with a woman (*k*  = 6, *estimate* = .12, *se* = .10, *z* = 1.11, *p* = .267, 95% CI [-.08, .32]). However, effect size variance could be explained by between study differences (*τ* = .21, *τ^2^* = .05, *I^2^* = 73.93%, *H^2^* = 3.84, *Q*_5_ = 16.99, *p* = .005). Including the moderator showed the cortisol change in intimate partner violence perpetrators was not different from young male students (*Q*_1_ = .86, *p* = .355; *estimate* = .26, *se* = .28, *z* = .93, 95% CI [-.29, .80]), see Figure S4.

*Excluding data current study* *raw cortisol values* Not including the data on the intimate partner violence perpetrators showed that cortisol levels did not change in response to contact with women (*k*  = 5, *estimate* = .16, *se* = .12, *z* = 1.38, *p* = .168, 95% CI [-.07, .39]). However, effect size variance could be explained by between study differences (*τ* = .22, *τ^2^* = .05, *I^2^* = 73.97%, *H^2^* = 3.84, *Q*_4_ = 13.76, *p* = .008). Inspection of the forest plot did not reveal any outliers. Evidence concerning the presence of a publication bias was mixed: there was no asymmetry in the funnel plot (*t*_3_ = .26, *p* = .812), but Duval and Tweedie’s trim and fill approach revealed that one study could be filled below the estimated effect size. Addition of this study resulted also in a non-significant change in cortisol levels in response to women (*estimate* = .10, *se* = .11, *z* = .84, *p* = .402, 95% CI [-.13, .32]).

**Figure S4:** Forest plot of all the studies measuring a raw cortisol change in response to contact with women.
